# Treatment of Exposed Pulps; and Criticism of Discussions before the Ohio State Dental Society

**Published:** 1875-05

**Authors:** J. S. King

**Affiliations:** Pittsburg, Pa.


### DENTAL REGISTER.

Vol. XXIX.] MAY, 1875. [No. 5.

### TREATMENT OF EXPOSED PULPS; AND CRITICISM OF DISCUSSIONS BEFORE THE OHIO STATE DENTAL SOCIETY.

BY J. S. KING, D. D. S., PITTSBURG, PA.

In looking over the January number of your interesting record, I was much interested in the perusal of the discussions that took place on Wednesday afternoon, December 3d, 1874, in the Ohio State Dental Society, during its late session held in the City of Columbus, Ohio. The disscussions I refer to were based upon the following proposition: "The Preservation of the Pulps of Teeth," subdivisions as follows: "*a.* Systemic conditions modifying treatment and influencing its results."

"*b.* The least injurious and yet efficient agents for covering and protecting the dental pulp and likewise promoting the formation of secondary dentine."

"*c.* Causes of failure."

In the remarks that followed the statement of the subject, with its interesting subdivisions, I was much interested. Also I was much amused by the generalizations indulged in by those participating in the discussions that took place on the subject. Dr. C. R. Butler, of Cleveland, in his opening remarks, gave expression to some trite thoughts and true sayings associated with the subject. Also Drs. Watt, Rehwinkel and Taft following Dr. Butler said many things good and true. Yet at times during the discussion each one seemed to be dancing to fancy time on the margins of the outer circle, which they seemed to have drawn around the subject under discussion. In those discussions, but little was evolved that can be regarded as practical, or that would lead the dental profession to adopt a mode of practice in the treatment of exposed tooth pulps. Beneficial, alike to themselves and to their patrons, so far as I could perceive, there was not a single presentation of a specific, or even of a general mode of practice in pulp treatment, by any one of the parties participating in the discussions, which would enable the enquiring dentist to take a step forward with any assurance of success in the all important operation of treating and preserving the vitality of exposed pulp tissues. Dr. Butler affirms that, "We can easily conceive of systemic conditions which would be unfavorable in the accomplishment of any thing like satisfactory results." This I admit may be true. Yet the admission or admonition need not carry with it any note of alarm, preventing the dental practitioner from immediately proceeding to treat and cover exposed vital pulp tissue of teeth whenever and wheresoever discovered.

Dr. Butler, in the course of his remarks, made use of the following, "There is a variety of modifying phases which are to be admitted and considered. In some cases we may almost abuse the pulp tissue, and yet it persistently retains its vitality; cut away a portion of it and even apply escharotics, as is the mistaken practice of some, (and I have known cases where arsenic has been applied) and yet the pulp has appeared almost entirely unaffected by the abuse. On the other hand, I have seen cases where the least exposure has been sufficient to destroy the pulp. So that we can only expect to arrive at anything like an intelligent course of practice to be pursued, by a careful observation of the structure of tissues involved also it is an abuse of the pulp, the temperament and organization of the patient to govern us in our efforts to restore to health and preserve the pulp." The thoughts contained in the above quotation are in some particulars correct. But in the use and application made of them, in these discussions on the subject under consideration, they are in many particulars incorrect. By means of the peculiar manner of their use the Doctor seems to have planted around his "intelligent course of practice" a formidable hedge, composed as he seems to think of very refractory materials, such as "structure of tissues involved the temperament and organization of the patient." These real or fancied conditions of our patients, be they physiological or pathological, are worthy of our observation; but so far as they relate to the subject of the treatment and preservation of the vitality of exposed pulp tissue of teeth, they are matters of mere secondary consideration, and should not for a moment prevent an immediate procedure on the part of the dental practitoner to cover any case of exposed pulp tissue placed under his care. Impliedly, the Doctor condemns the use of arsenic, characterizing its use as an abuse of the pulp. This position I heartily commend to the consideration of every member of the profession; "cutting away a portion of the pulp and even applying escharotics," he claims "Is the mistaken practice of some," and is an abuse. The cutting I admit is an abuse of the pulp; it is also an abuse to allow it exposed even for a moment, yet the necessities of the case will some times compel us to do both. In our text books, the classification of those remedies or agents called escharotics may be some what arbitrary, yet in the main they are correct.

But in discussing the subject of exposed pulp tissues and its treatment, it will not serve the best interests of our patrons nor yet of science, for us to condemn indiscriminately the use of a whole family of medicines, remedies or agents as a band of nuisances and outlaws simply because of their classification as being escharotics. Were we to call the given names of the whole family, perhaps we might justly condemn their use in the treatment of exposed pulp tissue, in the proportion of ninety-nine one-hundredths of the whole, yet the remaining one-hundredth may chance to be all that we could wish for in the accomplishment of our purposes. If a general fact of this character can be established by the experience of any given number of successful and reputable practitioners, then it becomes a duty to investigate by individual experiment the truth or falsity of such statements. In all cases of exposed vital pulp tissues of the teeth, it is correct practice to first cover the point of exposure; and secondly, to rightly diagnose and treat any existing pathological systemic conditions. This I conceive to be the ground or basis on which to discuss this subject of the treatment of exposed pulp tissue, utterly ignoring any given fancy in regard to individual temperaments, etc.

Dr. Watt, in his remarks upon the subject, said, "If you find only slight exposure and no pain, protect the pulp from extraneous matter soon as possible." On my basis of procedure we protect an exposed vital pulp from extraneous matter in all cases as soon as possible, whether the exposure be great or slight, whether accompanied with inflammatory action or pain, or without pain; and if the right materials are properly applied, all will do well, as in the case of the surgeon when caring for an ordinary wound. Dr. Taft's remarks on the subject seemed to be in unison with those preceeding. If the Doctor had been discussing the propriety and absolute necessity of a medico-dental education, his remarks would have been equally applicable and practical. He talks well without giving any data in regard to either a specific or general mode of treatment which would most likely be successful in any given case. He passes back and forth in the same groove marked out by those preceeding him in the discussion of the subject. He says, "To study as Dr. Watt has said, conditions and temperament, and endeavor to learn all about the peculiarities of the case, and what the requirements are, and then perform your operation in conformity with those requirements, constitutes the elements of success." These remarks of the Doctor just quoted, in some particulars, are undoubtedly true; but they are blinding counsel in the way they are given. They are simply learned phrases containing many of the elements of truth, but built upon a false basis, as may be seen in the following quotations from his subsequent remarks on the subject: "You hear many say, I treated a pulp so and so, without even reference to conditions, you may rest assured that he who talks on that wise does not recognize conditions, he has settled in a groove, and he runs back and forth continually, never leaving it, he gives his mode of treatment by successive steps, without making reference to systemic or local condition, thus indicating that he fails to recognise them, I predict that his operations will eventuate in failure nine cases out of ten.

These remarks just quoted may pass current as society discourse or as associational homily, but in their relations to the subject under consideration they will not answer for practical uses. Simply because they lead the dental practitioner into the error of taking first cognizance of systemic and local conditions, and treating the same, before proceeding to cover and thereby protect the exposed pulp tissue of teeth. If the form of the Doctor's argument had been to first cover the exposed pulp, and then treat systemic and local conditions, not even a single individual in the profession could justly interpose any objection to his position. As he then would be in harmony with all the facts and requirements associated with and pertaining to the important operation of treating exposed pulp tissue and thereby preserving the vitality of the pulp, providing that he accompanied the proposition with a detail of the materials or agents used, and the manner of their use. If Doctor Taft pursues a mode, method or manner of treating exposed pulps, wherein he is successful in three or four cases in five, excluding the average condition of exposed pulps and of patients that usually call for the services of the dental practitioner; why not detail his manner of proceeding? Perhaps his reply to this proposition can be discovered a little further on in the course of his remarks, where he seems to have evaded giving a direct answer to a question he so adroitly places in the mouths of his auditors, whose professional attainments and average intelligence has opened the way for them to a membership in the Ohio State Dental Society. The Doctor has just been urging the necessity of an honest endeavor on the part of the dentist, to change all the unfavorable conditions to a more favorable state when suddenly he placed his audience in the attitude of asking the following question: "What can I do?" The answer thereto comes from himself in the following words: "We can not have a course of lectures three or six months long to discuss the principles involved." I will accept this as his reply to the above proposition, yet I can not think the labor of three or six months was necessary on that occasion to so enlighten and instruct his auditors, (and others of equal ability and position) to that extent which would enable any one of them to successfully accomplish the simple yet invaluable operation of successfully covering exposed pulp tissue of human teeth, and thereby preserving the vitality of the same. Thus the Doctor flees from a satisfactory and practical response to his own interrogation, and continues his remarks in an endeavor to answer the following query: "Now what are some of the conditions which would modify treatment of the pulp?" In answer to this query the Doctor presents the following supposed systemic conditions, "Suppose then blood is in an impoverished condition." "Is deficient in some important and indispensable constituent, perhaps the patient is anæmic, or there is a deficiency of fibrin, or red or white corpuscles of the blood," or "The corpuscles may be defective and so be unable to perform their proper functions," and in addition to these varied pathological systemic conditions, he enumerates others where insidious poisons at work, instancing "malarial poisons" saying to his audience the following: "If you were going to select cases for treatment, you certainly would avoid those which you know were charged with malaria" Now any one or all of these enumerated pathological systemic conditions may possibly seem to be insuperable barriers to to immediate success in the treatment of exposed pulp tissue, so far as Doctor Taft's experience and that of the Ohio State Dental Society is concerned; but with those whose experiences prove the error of the position contended for in these discussions, these supposed unfavorable systemic conditions, either separately or collectively, would not in any degree prevent them from immediately proceeding to cover exposed pulp tissue of teeth when placed under their care, any more than the same conditions would prevent the operative surgeon from immediately proceeding to close an ordinary flesh wound or incision in the persons of the same class of patients. This being accomplished, they will as a secondary consideration, then proceed to give attention to and treat unfavorable systemic conditions, or which is more proper, refer the case to the family physician.

Dr. Rehwinkel remarks on the subject under consideration were as general and vague in their tone as were those of Drs. Butler, Watt and Taft. In so far as they related to "the least injurious and yet efficient agents for covering and protecting the dental pulp."

The Doctor sets out in his remarks by yielding a hearty concurrence to the remarks of Dr. Watt and then pursues the same general tone of argument. When speaking of mere exposure of the pulp he says: "If the pulp therefore is accidentally wounded, so far from feeling discouraged as to the final result of an attempt to save it, it would not disturb me in the least." This is a noble phrase; this language has in it the right ring, it is good doctrine, but why does the doctor follow these words with the following phrase: "I would be very careful however what agents I employed." And then so persistently refrain from even naming the "agents" he would "employ" under any given or supposed systemic conditions. Again he says, "I should think it perfect folly to put creosote upon a pulp which is in a healthy condition, Why? Because it is an escharotic." Does the Doctor in the use of the above language mean to say that he would put creosote upon an unhealthy pulp, if so, the force of such an argument would be that an unhealthy pulp is better able to bear the action of that escharotic, than is a healthy pulp, such an argument being fallacious, therefore, I will presume that he entirely ignores the use of creosote in pulp treatment; but can this be? For he says in another place "We would not think of using creosote in its full strength upon an ordinary wound in order to induce it to heal by first intention we might use it diluted to sponge out the wound." This last argument is that creosote diluted, is an agent that will promote or induce healing by first intention in an ordinary wound. If this be true, why will it not induce healing by first intention in the case of an ordinary wound in healthy pulp tissue? Again, the Doctor says, "We should employ the simplest treatment and mildest remedies" giving his reasons for so doing in the following language: "Because in the very elements of the blood we have recuperative power." In all candor I would ask what is "the simplest treatment" and what "the mildest remedy?" Why not name some of them and the manner of their use with a statement of the systemic conditions indicating their use? I admit that the Doctor has doubtingly expressed the thought that "Lacto-phosphate of lime is of value in simple and uncomplicated cases." As this agent has been used only about one year in this relation, the Doctor therefore seems to be somewhat chary in his semi-endorsement of the same. One of the remarkable things (as reported in the REGISTER) to which the doctor gave utterance was the following: "If you want to go further and assist the formation of secondary dentine we may employ escharoties." I ask what escharotics will assist in the formation of secondary dentine? Why are they not given by their appropriate names so that there need not be any possibility of mistake? Dr. Watt, in his remarks, stated that "some time ago" he had "a young lady" patient, "The perfect picture of health, which circumstance he thought was favorable to an attempt to save the pulp." Good health in this case was certainly the young lady's good fortune, as well as for the life of her tooth. However, the Doctor capped the pulp "with a shaving of Hill's stopping." Which subsequently he removed. When, lo! "There was a little button of secondary dentine protecting the pulp." In this case, the Doctor was frank enough to state what agent he used in capping the pulp. He did not claim however that Hill's stopping would assist in the formation of secondary dentine. Nor did he claim superiority for it over other agents in aiding and promoting the formatiom of secondary dentine. He seems only to have related simply an incident occuring in the course of his practice. Yet in this lone incident was concentrated, seemingly, the very acme of the Doctors wishes, a deposit of secondary dentine over the former exposed pulp. The argument seems to be, that unless the pulp becomes protected by secondary dentine, it cannot be saved. However, this particular case of Dr. Watt was certainly very remarkable, especially so, when we consider the youth and the certified good health, or systemic conditions of the patient. If in the stead of good health, the Doctor's patient had been the victim of exsanguinity or had been suffering from the effects of acute inflammatory rheumatism or of complicated neuralgic affections, in which cases, the deposit of secondary dentine is a thing of common occurrence in the form of hypertrophy or of exostosis upon the peripheral surfaces of roots of teeth, and sometimes in the form of nodules adhering to the inner walls of the pulp chamber, where it is considered to be, and is truly a pathological result. Therefore in this particular case, where it was most unlikely to occur, it was certainly somewhat anomolus, and worthy the notice and attention the Doctor gave it. Now if secondary dentine is a good thing in those points or localities where it may be useful, and if there are any known escharotics as intimated by Dr. Rehwinkel, that will assist in the formation thereof, why did not the Doctor in consideration of his professional relations distinctly name them, and set forth the proper method of their use, with an account or statement of the local or systemic conditions indicating the propriety of their use.

Dr. Butler says, that the use of escharotics, "is the mistaken practice of some." There was evidently in some points a conflict of opinion among the savants. Whilst the others (the majority perhaps) they seemed to harmonize wonderfully.

Dr. Rehwinkel, near the close of his remarks, said that: "The great secret of success is not to over do." Also he says that, "the causes of failure can be summed up as follows: want of skill in manipulation, want of judgment in selecting remedies, etc., and above all in doing to much." There is but a little doubt but that the failure to do, as well as that of overdoing will often result in disaster, so far as I can perceive, judging as I do from the REGISTER's report of the discussions occurring in the Ohio State Dental Society, the success attained by the society in the discussion of the subject was in but one direction; the extreme necessity of a medical education in order to be qualified or enabled to properly and successfully treat exposed pulp tissue of the teeth. Their failure consisted in doing too little, in the way of pointing out the remedies or agents that should be used in the pulp treatment and the manner of their use, whether used in accordance with given systemic conditions, or regardless of such conditions I ask did they not fail in the discharge of their duties to the public at large (inasmuch as the Ohio State Dental Society is one of the acknowledged lights in the profession in said state) in not commending or indicating in any manner a method or system of treatment, that would promise even a degree of success in the hands of any one possessed of average skill and attainments professionally considered? Excepting only their laudations of qualifications and endowments to be derived from a medical education. But in this instance, even this qualification has signally failed to enlighten us on the best or "The least injurious and yet efficient agents for covering and protecting the dental pulp."

My position on the subject of pulp treatment has been quite plainly indicated in the foregoing pages of this paper. As plainly as I can do so, I will now proceed to state my system of treating exposed pulp tissue, and the remedies or agents I use. In all cases of exposed pulps placed under my care, I immediately proceed to cleanse the cavity containing the exposure as thoroughly as though I were at once to proceed and fill the same with gold. This done, I then proceed to cover the pulp immediately, without regard to constitutional idiosyncrasies or tendencies or peculiarities of temperament or of any general unfavorable systemic conditions nor do my "operations eventuate in failure nine cases out of ten." The "efficient agents" I make use of are creosote, oxide of zine and liquid chloride of zinc. The manner of their use is as follows: Upon a glass or porcelain slab, place a small parcel of oxide of zinc mix the same to consistency of a stiff paste by adding to it creosote with this paste lightly cover the exposed pulp. It is absolutely necessary that the entire exposure be covered with the creosote paste, and that this covering extend some little distance past the borders of the orifice of exposure, this covering now being in place and not allowed to spread out over the side walls of the cavity, but strictly confined to its proper limit, is now ready for the second or outer covering. I then proceed to mix (upon the same or separate slab) to the consistency of a soft paste oxide of zinc with liquid chloride of zinc (this is commonly called oxychloride of zinc) as quickly as possible, I place this second mixture in the cavity, being careful not to displace my first covering, thus I fill the cavity to the desired fullness. If the exposure has been unaccompanied with pain, I proceed immediately to cut away sufficient of the outer covering of oxychloride to enable me at once to proceed to insert thereon a good gold filling. But if the pulp has been largely exposed or has been painful. I dismiss my patient for ten days or longer, and subsequently finish the operation with gold. Occasionally in the more aggravated cases of exposure it becomes necessary to remove the covering and renew the same on account of unsubdued inflammatory action in the pulp tissue, or on account of failure in not properly covering the pulp this I am pleased to say is of rare occurrence. Occasionally, a pulp may be covered at a point in time or in its condition where the vital forces have just ceased their normal action, or in other words just at that point where vitality becomes extinct. If so, trouble will inevitably follow such a condition, this system of treatment has been remarkably successful in my practice, and equally as successful in the practice of others, to whom I have communicated my method of treatment.

My paper has already doubled the limit I had in comtemplation when I took up my pen to write this article, yet I felt that justice requires that I give in this article a synopsis of a report handed me some ten days ago by my friend and co-laborer in the profession, W. F. Fundenberg, M. D., for many years a practicing dentist in this city, and brother-in-law to the late S. P. Hulihen, M. D., of Wheeling, W. Va. Two years and four months ago I communicated to Dr. Fundenberg my method of treating pulp exposures, requesting him to try some experiments in my method of treatment, and to keep a just and true record of the cases he might treat, so far as he could asertain the facts in relation thereto; this he claims to have done, and the following is a synopsis of his report:

Whole number of pulp exposures covered in a period of two years and three months—one hundred and three. Those that were known to be successful up to February 1st, 1875, were seventy-one. Those not known and from whom a report was not received twenty-six, those that were known to be failures were six, three of these six failures occurred in the first six cases treated by Dr. Fundenberg.

This report of the experience of Dr. Fundenberg in my mode of treatment is certainly encouraging. It is not an isolated experience; there are others pursuing the same system of treatment who are equally successful, all of whom are regardless of constitutional idiosyncrasies, or of real or fancied differences or peculiarities in temperament and organization of systemic conditions, except only as matters of secondary consideration, to be properly cared for when the first duties of the dentist have been attended to and properly performed.

